# Identifying drug targets for schizophrenia through gene prioritization

**DOI:** 10.1038/s41398-026-03813-0

**Published:** 2026-02-04

**Authors:** Julia Kraft, Alice Braun, Swapnil Awasthi, Georgia Panagiotaropoulou, Marijn Schipper, Nathaniel Bell, Danielle Posthuma, Antonio F. Pardiñas, Stephan Ripke, Karl Heilbron

**Affiliations:** 1 Department of Psychiatry and Psychotherapy, Charité – Universitätsmedizin Berlin, Berlin, Germany; 2 Stanley Center for Psychiatric Research, Broad Institute of MIT and Harvard, Cambridge, MA USA; 3 German Center for Mental Health (DZPG), partner site Berlin/Potsdam, Berlin, Germany; 4 Vrije Universiteit Amsterdam, Amsterdam, The Netherlands; 5 Department of Child and Adolescent Psychiatry and Pediatric Psychology, Section Complex Trait Genetics, Amsterdam Neuroscience, Vrije Universiteit Medical Center, Amsterdam, The Netherlands; 6 Centre for Neuropsychiatric Genetics and Genomics, Division of Psychological Medicine and Clinical Neurosciences, Cardiff University, Cardiff, UK; 7 Present Address: Bayer AG, Research & Development, Pharmaceuticals, Berlin, Germany

**Keywords:** Genetics, Drug discovery, Schizophrenia

## Abstract

Schizophrenia genome-wide association studies (GWASes) have identified >250 significant loci and prioritized >100 disease-related genes. However, gene prioritization efforts have mostly been restricted to locus-based methods that ignore information from the rest of the genome. To more accurately characterize genes involved in schizophrenia etiology, we applied a combination of highly-predictive tools to a published GWAS of 67,390 schizophrenia cases and 94,015 controls. We combined both locus-based methods (fine-mapped coding variants, distance to GWAS signals) and genome-wide methods (PoPS, MAGMA, ultra-rare coding variant burden tests). We extracted genes that 1) are targeted by existing drugs that could potentially be repurposed for schizophrenia, 2) are predicted to be druggable, or 3) may be testable in rodent models. We prioritized 101 schizophrenia genes, including 15 that are targeted by approved or investigational drugs (*e.g*., *DRD2, GRIN2A*, *CACNA1C, GABBR2*). Of these, 7 have never been tested in clinical trials for schizophrenia or other psychiatric disorders (*e.g., AKT3*). Seven genes are not targeted by any existing small molecule drugs, but are predicted to be druggable (*e.g., GRM1*). We prioritized two potentially druggable genes in loci that are shared with an addiction GWAS (*PDE4B* and *VRK2*). We curated a high-quality list of 101 genes that likely play a role in the development of schizophrenia. Developing or repurposing drugs that target these genes may lead to a new generation of schizophrenia therapies. Rodent models of addiction more closely resemble the human disorder than rodent models of schizophrenia. As such, genes prioritized for both disorders could be explored in rodent addiction models, potentially facilitating drug development.

## Introduction

Schizophrenia is a highly-heritable and heterogeneous disorder characterized by positive symptoms (*e.g*. delusions and hallucinations), negative symptoms (*e.g*. blunted affect), and cognitive impairment [1]. Schizophrenia patients are often also diagnosed with neurodevelopmental disorders [1, 2] (*e.g*. intellectual disability and autism spectrum disorder) and other psychiatric conditions [3, 4] (*e.g*. substance use disorder [SUD] and depression). Antipsychotic medications antagonizing the dopamine receptor D2 are currently the first-line treatment for schizophrenia. However, approximately 34% of patients are considered treatment-resistant [5], and especially cognitive deficits and negative symptoms often persist [6, 7]. These unmet clinical needs, as well as the high burden of antipsychotic side effects [8, 9], clearly underline the necessity for pharmacotherapies with novel mechanisms of action.

Only 6.2% of psychiatric drug programs that enter Phase I trials are ultimately approved—well below the average success rate of 9.6% across all medical areas [10]—and investment in psychiatric drug development programs have decreased in recent years [11]. This low success rate likely reflects the complex nature of mental disorders, limited knowledge of disease mechanisms, and sparsity of validated animal models. Given that 63% of drugs approved by the FDA from 2013–2022 were supported by human genetic evidence [12], pursuing targets that are genetically-linked to disease may lead to increased success rates [13, 14]. A major source of this human genetic evidence comes from genome-wide association studies (GWASes) [13, 14]. For instance, schizophrenia GWASes [15–17] have identified a robust association near *DRD2*, which encodes the dopamine receptor D2. It is estimated that only 1.9% of genetically-supported drug targets for psychiatric disorders have been clinically explored [18], suggesting that follow up of other schizophrenia GWAS findings may eventually lead to the design of new medicines.

Several studies have attempted to prioritize the causal genes underlying published schizophrenia GWAS loci using a variety of methods, including: expression quantitative trait loci mapping and transcriptome-wide association studies [19–21], massively parallel reporter assays [22], summary data-based Mendelian randomization (SMR) [17, 23], and Hi-C-coupled MAGMA [24]. The largest published schizophrenia GWAS identified 287 significant loci and prioritized 120 genes for follow up using fine-mapped credible sets [25], SMR [26], and Hi-C interactions between enhancers and promoters [27]. However, a recent study has found that these methods vary substantially in their precision to correctly predict “probable causal genes” (defined using fine-mapped coding variants) [28]. They found that only 29% of genes with the lowest SMR P-value in their locus were probable causal genes, while this proportion was even lower (~14%) for genes with the strongest promoter capture Hi-C evidence.

Here, we prioritized genes likely to play an important role in schizophrenia (SCZ) risk using high-precision prioritization methods: 1) polygenic priority score (PoPS) combined with nearest gene, 2) fine-mapped coding variants, and 3) and ultra-rare coding variant burden tests [29]. We nominated 101 genes, 15 of which are targets of approved drugs (10 genes) or drugs that have been tested in clinical trials (“investigational drugs”, 5 genes). We discuss the potential utility of these drugs for treating schizophrenia and highlight an additional 7 prioritized genes that may be tractable via small molecule drugs.

## Methods and materials

### Ethics statement

This research was conducted in accordance with the ethical standards of the institutional and national research committees. Informed consent was obtained from all participants. Details on Institutional Review Board approvals of the individual studies included in the presented work are provided in the original publication [17].

### GWAS summary statistics

We analyzed the publicly-available “core dataset” of GWAS summary statistics from the largest published SCZ GWAS from the Psychiatric Genomics Consortium (hereafter we will refer to this study as “PGC3”) [17], a meta-analysis of 90 cohorts of European (EUR) and East Asian (EAS) descent including 67,390 cases and 94,015 controls (effective sample size [N_eff_] = up to 156,797). For analyses requiring data from a single ancestry, we used the EUR subset of the core dataset (76 cohorts, 53,386 cases, 77,258 controls, effective sample size [N_eff_] = up to 126,282) and the EAS-ancestry subset (14 cohorts, 14,004 cases, 16,757 controls, N_eff_ = up to 30,515).

### Reference panels

Accordingly, we used external data from the Haplotype Reference Consortium release 1.1 (HRC) to construct three linkage disequilibrium (LD) reference panels: an EUR panel (*N* ≥ 16,860), an EAS panel (*N* ≥ 538), and an EUR + EAS panel that included both EUR and EAS individuals in the same proportions as the GWAS summary statistics—80% EUR and 20% EAS (*N*_EUR_ = 2,191, N_EAS_ = 538).

### Variant quality control

We removed EUR + EAS GWAS variants with: 1) a minor allele count < 10 (minor allele frequency [MAF] < 0.0018) in the EUR + EAS reference panel (259 variants removed), 2) a reported allele frequency that differed from the reference panel frequency by > 0.1 (29 variants removed), and 3) a reported allele frequency that differed from the reference panel frequency by > 12-fold (11 variants removed). After quality control, 7,584,817 variants remained.

### Isolating independent association signals

In order to disentangle statistically-independent genetic signals in the EUR + EAS dataset, we first clumped variants using PLINK v1.9 [30] (*P* < 5×10^-8^, *r*^2^ < 0.1, window size = 3Mbp) and our EUR + EAS reference panel, expanded the boundaries of each clump by 500 kb on either side, and merged overlapping boundaries. Within each resulting region, we ran COJO [31] and removed hits with joint *P* > 5×10^-8^. If multiple independent hits in a region were found, we used COJO to isolate each signal by performing leave-one-hit-out conditional analysis. For each isolated signal, we computed credible sets (CSs) using the finemap.abf function in the coloc R package [32, 33]. Finally, we defined loci as ±300 kb around each credible set.

### MAGMA

We performed gene-based association tests using MAGMA [34] (“SNP-wise mean model”) and all variants with MAF > 1%. We analyzed the EUR- and EAS-based GWASes separately using the corresponding ancestry-specific reference panel and MAFs. We mapped variants to protein-coding genes using Genome Reference Consortium Human Build 37 (GRCh37) gene start and end positions from GENCODE v44 [35]. We removed genes that had fewer than 3 variants mapped to them. For each gene, we meta-analyzed the resulting ancestry-specific MAGMA z-scores weighted by the square root of sample size [36].

### PoPS

MAGMA z-scores can be thought of as a proxy for the magnitude of effect that each gene has on schizophrenia risk. As such, PoPS [28] seeks to learn the properties of schizophrenia risk genes by training a ridge regression model to predict MAGMA z-scores using more than 57,000 gene-level features. These include 40,546 features derived from bulk and single-cell gene expression datasets (*e.g*., principal components of expression and cell type-specific expression), 8,718 features derived from predicted protein-protein interaction networks from InWeb_IM, and 8,479 features derived from curated biological pathways (*e.g*., the gene ontology and Reactome databases [37, 38]). PoPS output values are the fitted values of this ridge regression model, and represent the MAGMA z-score that a gene is predicted to have based on its properties. The original PoPS study recommended selecting the gene with the largest PoPS value in the locus rather than using a specific numerical threshold [28]. Using the ancestry-specific MAGMA results as input, we performed PoPS [28] using all 57,543 gene-based features as predictors. These features were not available for chrX so we restricted our analysis to autosomal genes. The resulting ancestry-specific PoPS values were then also meta-analyzed weighted by the square root of sample size. We only used the meta-analyzed MAGMA and PoPS values for gene prioritization.

This same study introduced a novel tool for gene prioritization, the polygenic priority score (PoPS). By intersecting genes with the top PoPS value in their locus with genes that were nearest to their GWAS signal, they were able to achieve a precision of 79%.

### Gene prioritization criteria

Following the original PoPS publication, we prioritized genes that met both of the following criteria: 1) had the top PoPS value in a given locus and 2) were the nearest gene to the corresponding GWAS signal based on the posterior inclusion probability (PIP)-weighted average position of credible set variants. Under these criteria, however, it is possible that the top POPS value within a locus is relatively weak on a genome-wide scale, or that the nearest gene is nevertheless relatively distant. We therefore also required that genes have a PoPS value in the top 10% of all values genome-wide. We also prioritized genes that had 1) PIP > 50% for non-synonymous credible set variants affecting the gene, or 2) false discovery rate-corrected P value (P_FDR_) < 5% in a published SCZ burden test of ultra-rare coding variants [29]. We used non-synonymous variants from the “baseline-LF 2.2.UKB model” (80,693 variants) and subsetted to those with an estimated per-variant heritability > 1×10^-7^ (removed 4,709 variants, all with estimated h^2^ < 1×10^-10^: >1,000-fold smaller) [39]. We removed loci that contained more than 20 genes since larger loci are more challenging to resolve [40], but we have included results for these large loci in Table S3.

### Comparison with previous schizophrenia gene prioritization efforts

We compared our prioritized genes with those highlighted in the original PGC3 publication. Specifically, we extracted the “Symbol.ID” and “Prioritised” columns from Table S12. While the PGC3 study utilized the same core dataset, they restricted analysis to loci that retained genome-wide significance in the “extended GWAS”—a meta-analysis of the core dataset, 9 cohorts of African American and Latin American ancestry, and a dataset from deCODE genetics. They prioritized genes using a combination of FINEMAP, SMR, Hi-C interaction mapping, and non-synonymous or untranslated region credible set variants with PIP > 10%. The PGC3 study validated their list of prioritized genes by looking for overlap with genes expressed in brain tissue, genes with signatures of mutation intolerance in large-scale exome studies [41], or genes linked to schizophrenia through rare genetic variation in the SCHEMA study [29]. Furthermore, they also found genetic overlaps in other neurodevelopmental conditions using sequencing studies from autism spectrum disorder [42] and developmental disorder [43]. We incorporated a subset of this information by extracting the “ASD” and “DDD” columns from Table S12 of the PGC3 study. For full details, please refer to the original publication [17].

### PsyOPS

We further validated our prioritized genes using the Psychiatric Omnilocus Prioritization Score (PsyOPS) tool [44]. The original PsyOPS publication [44] found that PsyOPS achieved similar performance to PoPS in predicting causal psychiatric disease genes, but using only three predictors: probability of loss-of-function intolerance (pLI) > 0.99, brain-specific gene expression, and overlap with 1,370 known genes for neurodevelopmental disorders (autism, epilepsy, intellectual disability). PsyOPS treats the nearest gene to each GWAS hit as a proxy for the causal gene in the locus, trains leave-one-chromosome-out logistic regression models, and outputs the predicted probability that a given gene is causal. We determined a gene to be prioritized by PsyOPS if the predicted probability of being a causal gene exceeded 50%. We computed PsyOPS scores using all 257 independent schizophrenia GWAS hits.

### Drug target mapping

We determined whether our prioritized genes were targeted by approved or investigational drugs using GraphQL API queries of the Open Targets platform [45], which in turn queries the EMBL-EBI ChEMBL database. For genes that were not targeted by approved or investigational drugs, we performed additional Open Targets API queries to extract evidence of drug tractability—the probability of identifying a drug that is able to bind and modulate a given target. We focussed on small molecule drugs, but results for other modalities can be found in Figure S1.

### Colocalization with other studies

We prioritized several genes that have also been highlighted by recent GWASes for addiction [46] and Parkinson’s disease [47]. Using the EUR reference panel, we processed EUR-ancestry GWAS summary statistics from these studies using the same pipeline described above. We identified loci that physically overlapped with schizophrenia loci and computed the posterior probability of colocalization (H_4_) using all variants in the shared locus and the coloc.abf function in the coloc R package [32, 33].

## Results

**Fig. 1:**
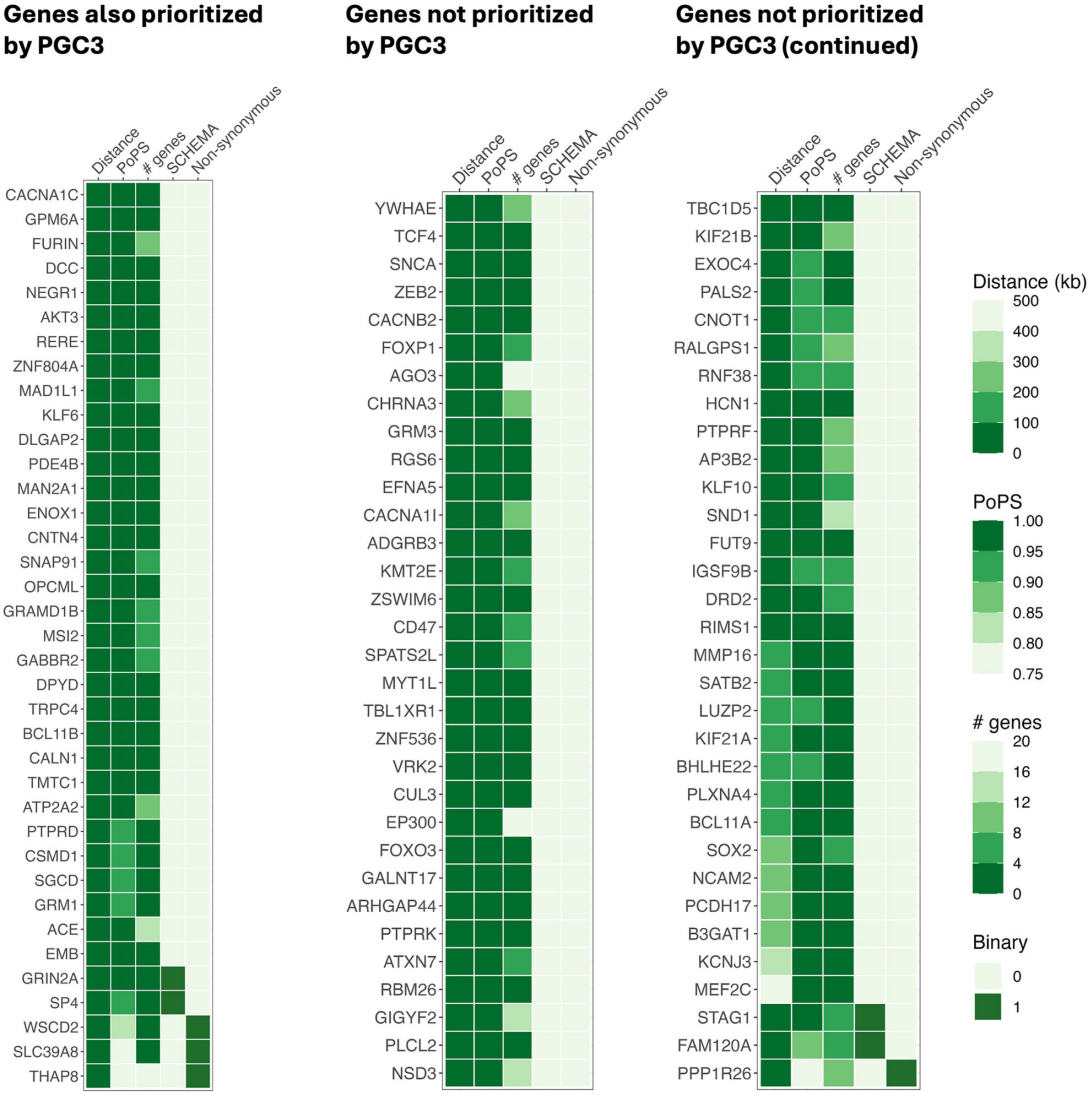
Heatmap. An overview of the evidence supporting each prioritized gene, separated based on whether they were (left panel) or were not (middle and continued in the right panel) previously prioritized in the PGC3 study [17]. Distance: distance in kilobases between gene and credible set. PoPS: PoPS percentile where 0 represents the smallest genome-wide value and 1 represents the largest. # genes: number of genes in the locus. SCHEMA: a binary indicator of whether ultra-rare coding variant burden in a given gene was also significantly associated (P_FDR_ < 5%) with schizophrenia in a study from the Schizophrenia Exome Sequencing Meta-analysis (SCHEMA) consortium [29]. Non-synonymous: a binary indicator of whether the credible set contained non-synonymous variants with a summed posterior inclusion probability >50%. Genes are sorted first by “Non-synonymous”, then by SCHEMA, distance, and PoPS percentile.

**Fig. 2:**
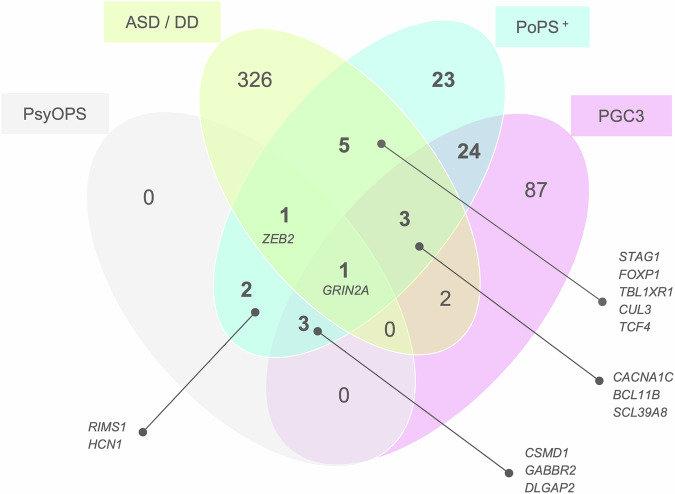
Venn diagram. Venn diagram showing the overlap between the number of genes identified by the present analysis (PoPS^+^), rare-variant studies of autism spectrum disorder (ASD) and/or developmental disorder (DD), the Psychiatric Omnilocus Prioritization Score (PsyOPS), and prior gene prioritization efforts (PGC3). Gene symbols are displayed for a subset of intersecting regions.

We prioritized schizophrenia genes using the “core dataset” from the largest published schizophrenia GWAS meta-analysis [17], “PGC3” (67,390 cases and 94,015 controls). We identified 257 independent associations with *P* < 5×10^-8^ (Table S1). Across these loci, we prioritized 101 schizophrenia genes (Fig. 1, Table S2) based on their distance to the credible set, PoPS values, number of genes in the locus, presence of non-synonymous variants in the credible set, and support from a published schizophrenia burden test of ultra-rare coding variants [29] (see Methods). To corroborate our findings with convergent evidence, we compared them with prioritization efforts from the PGC3 study [17], genes linked to autism spectrum disorder [42] (ASD) and developmental disorder [43] (DD) via sequencing studies, and results from the PsyOPS tool (Fig. 2). Across all genes in GWAS loci, prioritized genes were also DD and/or ASD genes (Fisher’s exact test P = 7.8×10^-11^, odds ratio [OR] = 11) or PsyOPS genes (Fisher’s exact test P = 1.7×10^-8^, OR = 18) significantly more often than expected due to chance.

### Overlap with previous schizophrenia gene prioritization efforts

Of our 101 prioritized genes, 37 (37%) were also prioritized in the PGC3 study (“overlapping genes”) and several sources of evidence suggest that these genes are likely to play a role in schizophrenia risk. Ultra-rare coding variant burden in two overlapping genes (*GRIN2A* and *SP4*) was significantly associated (P_FDR_ < 5%) with schizophrenia in the SCHEMA study [29]. Similarly, five overlapping genes (*GRIN2A, CACNA1C*, *BCL11B*, *RERE*, and *SLC39A8*) were also identified by rare variant exome sequencing studies of DD [43] and/or ASD [42] (see Fig. 2). Furthermore, the lead schizophrenia variant in the *SLC39A8* locus is a non-synonymous variant (PIP = 99%) that has been investigated in detail elsewhere [48]. *WSCD2* (PIP = 53%) and *THAP8* (PIP = 88%) were also prioritized due to a non-synonymous variant in the credible set. Four overlapping genes (*GRIN2A*, *DLGAP2*, *GABBR2*, and *CSMD1*) were nominated by PsyOPS (see Methods). Notably, *CSMD1* is known to inhibit the complement cascade, has reduced expression in first-episode psychosis patients [49], and knockout mice have exhibited behaviors resembling schizophrenia negative symptoms [50].

### Genes that were not nominated by previous schizophrenia gene prioritization efforts

**Fig. 3:**
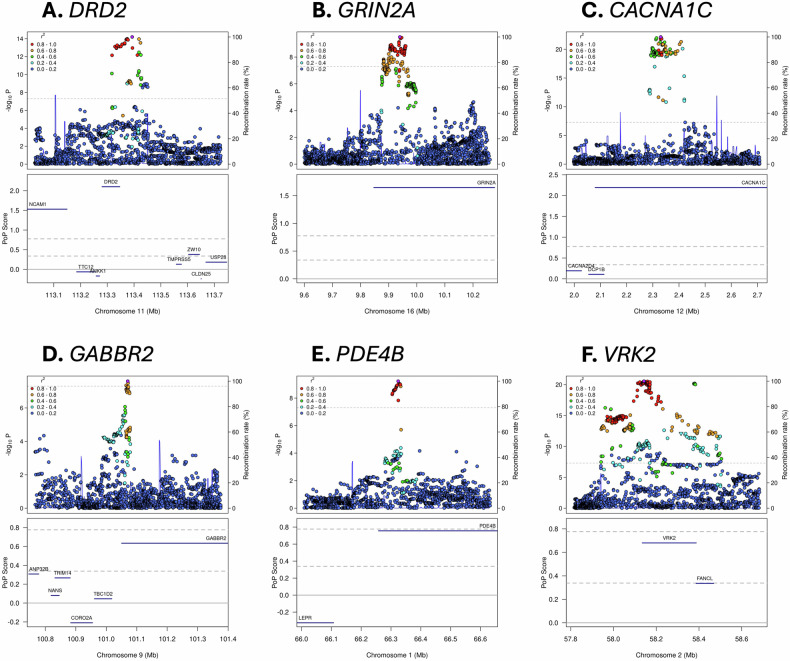
Variant-level associations and PoPS results for selected loci. The prioritized genes in plots **A-E** are targets of approved drugs; the prioritized genes in plots **E**, **F** are in loci shared by an addiction GWAS [46]. The upper portion of each sub-plot is a LocusZoom plot. Each point represents a different genetic variant, the x-axis represents physical position on the listed chromosome, the left y-axis represents –log_10_-transformed P value, the right y-axis represents the recombination rate, colour represents linkage disequilibrium with the lead variant in the locus (as shown in the legend), and the horizontal dashed line represents the genome-wide significance P value threshold of 5×10^-8^. The lower portion of each figure is a PoPS plot. Genes are denoted as blue bars spanning from their transcription start site to their transcription stop site using the same x-axis as the LocusZoom plot, the y-axis represents the raw PoPS score, the dashed horizontal grey lines represent the top 10% and 1% of PoPS scores genome-wide, and the solid horizontal grey line represents a PoPS score of 0.

Of our 101 prioritized genes, 64 (63%) were not prioritized in the PGC3 study (“non-overlapping genes”). Two non-overlapping genes (*STAG1* and *FAM120A*) were significantly associated with ultra-rare coding variant burden in the SCHEMA study [29]. Twelve non-overlapping genes (*STAG1*, *ZEB2*, *MYT1L*, *TBL1XR1*, *SATB2*, *MEF2C*, *CUL3*, *BCL11A*, *KMT2E*, *FOXP1*, *EP300*, and *TCF4*) were also identified by rare variant exome sequencing studies of DD [43] and/or ASD [42]. Note that *TCF4* was not prioritized in the PGC3 study because they only investigated regions containing three independent genetic associations or fewer and there were four associations near *TCF4*. We prioritized *PPP1R26* due to a non-synonymous variant in the credible set (PIP = 96%). Six non-overlapping genes (*ZEB2*, *MYT1L*, *SATB2*, *HCN1*, *RIMS1*, and *CACNA1I*) were nominated by PsyOPS (see Methods). Perhaps most importantly, our analysis highlighted the dopamine receptor gene *DRD2*, which is targeted by approved antipsychotic medications with varying affinity [51] (Fig. 3A). Finally, 31 of our 101 prioritized genes (31%) were not prioritized by PGC3, but have been highlighted by recent studies that prioritized schizophrenia genes using gene co-expression [52, 53], expression quantitative trait loci mapping or TWAS [20, 54], epigenome-wide association study [55], massively parallel reporter assays [56], or 3D genome architecture analysis [57] (*e.g*., *AP3B2*, *CACNB2*, *CNOT1*, *MEF2C*, *RBM26*, *SATB2;* see Supplemental Material; Table S2).

### East Asian-specific gene prioritization

Because East Asian-ancestry individuals were underrepresented in our dataset relative to European-ancestry individuals, we tested whether applying our prioritization criteria to the East Asian cohort would identify additional ancestry-specific genes. There were eight genome-wide significant loci in the East Asian-only dataset. In these loci, we prioritized four genes: two that were also found in the multi-ancestry meta-analysis (*VRK2* and *SPATS2L*) and two that were specific to the East Asian dataset (*FRY* and *PTN*). The *FRY* locus was not genome-wide significant in the multi-ancestry meta-analysis. Although the *PTN* locus was genome-wide significant in the multi-ancestry meta-analysis and *PTN* had the top local PoPS value, it was not the nearest gene and was therefore not prioritized.

### Drug target mapping

In addition to *DRD2*, we prioritized 15 genes that are targeted by approved (10 genes) or investigational drugs (5 genes, Table S4, Table 1). Of these, 8 were also prioritized in the PGC3 study (*GRIN2A*, *CACNA1C*, *PDE4B*, *GABBR2*, *AKT3*, *DPYD, ACE*, and *ATP2A2*) and 7 (*CACNB2*, *GRM3*, *SNCA, CACNA1I, CD47, KCNJ3*, *and CHRNA3*) were prioritized in our analysis but not in PGC3 (see Discussion). Our list of prioritized genes also included 7 genes (*HCN1*, *VRK2*, *TRPC4, EP300, GRM1, PTPRF*, and *MMP16*) that belong to known druggable protein families [58] and are reported to bind to at least one high-quality ligand [45], suggesting potential as small molecule drug targets (Figure S1).

**Table 1:** Prioritized genes targeted by approved or investigational drugs, or nominated by tractability analysis.

Level of evidence	Genes
Clinical trial, schizophrenia	*DRD2*^A^, *GRIN2A*^A^*, CACNA1C*^A^*, GRM3, CACNA10I*^A^*, PDE4B*^A^
Clinical trial, other psychiatric disease	*GABBR2*^A^*, CHRNA3*^A^*, CACNB2*^A^*, ACE*^A^
Clinical trial, non-psychiatric disease	*CD47, ATP2A2, KCNJ3*^A^*, AKT3*^A^*, DPYD, SNCA*
Predicted to be tractable	*HCN1*, *VRK2*, *TRPC4, EP300, GRM1, PTPRF, MMP16*

^A^ – targeted by approved drugs.

## Discussion

We prioritized 101 genes near 257 independent GWAS signals. Of these genes, 52 (51%) were also supported by evidence (Fig. 2) from the PGC3 study (37 genes), DD/ASD sequencing studies (17 genes), and PsyOPS (10 genes). We prioritized *DRD2* (Fig. 3A) [51], as well as 15 other genes targeted by approved drugs (10 genes) or drugs that have been tested in clinical trials (5 genes). Of these, only 8 genes were targeted by drugs that have been trialed in schizophrenia or other psychiatric disorders (see Table 1).

Some of these druggable genes have also been highlighted by earlier drug repurposing studies, including genes that are targeted by drugs affecting calcium signaling (*CACNA1C* [19, 59–61]*, CACNA1I* [60, 61]*, CACNB2* [59, 60]), glutamatergic *(GRIN2A* [19, 59, 60]*, GRM3* [59, 61]), GABAergic (*GABBR2* [60]) and cholinergic pathways (*CHRNA3* [60]). Our analyses do not predict whether the effect of these drugs (*e.g*. inhibitor) aligns with the effect that would be desired for schizophrenia. Therefore, we will now discuss literature supporting the potential for these drugs to be repurposed as treatments for schizophrenia. We also identified 7 other genes that may represent tractable small molecule drug targets and discuss how additional target validation could stimulate drug development programs.

### Glutamate receptors: *GRIN2A*, *GRM3*, and *GRM1*

We prioritized *GRIN2A*, which encodes a subunit of the N-methyl-D-aspartate receptor (NMDA-R, Fig. 3B). In addition to GWAS, there is evidence that decreased NMDA-R function increases schizophrenia risk from *GRIN2A* ultra-rare variant burden tests [29], *GRIN2A* mouse knockout models [62], and pharmacological antagonism of the NMDA-R [63]. This raises the possibility that increasing NMDA-R activity may provide therapeutic benefit for schizophrenia patients. A meta-analysis of 4,937 schizophrenia patients from 40 randomized controlled trials found that NMDA-R modulator augmentation (*e.g*. via glycine or glycine transporter type I inhibitors) significantly improved total, positive, and negative schizophrenia symptoms versus placebo [64]. These compounds have also been proposed as a therapeutic strategy for schizophrenia patients who are treatment-resistant or have impaired cognition [65]. There are currently three Phase III clinical trials underway assessing the effect of iclepertin, a glycine transporter type I inhibitor, on cognitive impairment associated with schizophrenia [66]. If ultimately approved, this may become the first medication indicated to treat the cognitive symptoms of schizophrenia.

We also prioritized *GRM3*, which encodes a different glutamate receptor: metabotropic glutamate receptor 3 (mGluR_3_). Clinical trials of pomaglumetad methionil, an mGluR_2/3_ agonist, have yielded inconclusive effects on positive symptoms [67–71]. However, an analysis of clinical trial data suggested that specific patient subgroups may have benefited [72] and preclinical research has suggested that a cognitive endpoint may be more appropriate [73, 74]. Similarly, positive modulation of mGluR_1_ (encoded by *GRM1*) showed pro-cognitive effects in animal studies (*e.g*., reversal of working memory deficits) possibly via interneuronal regulation of cortical inhibition [75]. MGluR1 activation also decreases striatal dopaminergic neurotransmission, which could lead to antipsychotic-like properties (*e.g*., reduction in positive symptoms) [76]. Interestingly, the newly approved schizophrenia drug, KarXT, attenuates dopamine release and exerts antipsychotic-like effects by activating M4 muscarinic acetylcholine receptors [77, 78]. This process appears to be codependent on mGluR1 activation [77].

### Voltage-gated calcium channels: *CACNA1C*, *CACNB2*, and *CACNA1I*

We prioritized *CACNA1C* (Fig. 3C), which encodes the alpha-1 subunit of a voltage-gated calcium channel (Ca_v_1.2). A Phase III clinical trial for bipolar disorder showed that 11 out of 13 non-responders to first-line therapy (lithium) showed a clinically-meaningful response to verapamil (a calcium channel blocker [CCB]), or verapamil + lithium [79]. The genetic correlation between schizophrenia and bipolar disorder is approximately 70% [2] and a recent bipolar disorder GWAS also identified a significant association near *CACNA1C* [80], suggesting that verapamil may be a promising treatment option for schizophrenia. Other CCBs may also be effective—a large cohort study (N = 10,460) found that use of dihydropyridine CCBs was associated with reduced risk of psychiatric rehospitalization [81]. CCBs may also improve certain cognitive functions [82, 83]. The use of CCBs for treating schizophrenia is further supported by the fact that we prioritized *CACN2B*, an auxiliary subunit of voltage-gated calcium channels. A T-type calcium channel antagonist targeting Ca_v_3.3 (encoded by *CACNA1I*) has been tested in schizophrenia patients in a phase II trial, which produced inconclusive evidence since neither the investigated drug (MK-8998) nor the active comparator (olanzapine) showed significant symptom improvement [84].

### Loci shared with addiction: *PDE4B* and *VRK2*

We prioritized *PDE4B*, which encodes phosphodiesterase 4B (Fig. 3E). A recent GWAS of an addiction-related latent factor derived from four SUDs [46] also found a signal near *PDE4B* and highlighted *PDE4B* as the likely causal gene. SUDs are frequently comorbid with schizophrenia [4] and there is significant genetic correlation between schizophrenia and several SUDs [85]. While it is challenging to assess psychotic symptoms in rodents, high-quality rodent addiction models exist for a wide range of substances [86]. Indeed, several drugs that are approved to treat alcohol use disorder (*e.g*. naltrexone and acamprosate) were originally pursued based in part on success in preclinical animal models [86, 87]. Administering ibudilast, a drug that inhibits *PDE4B* and other phosphodiesterases, has been shown to reduce alcohol intake by approximately 50% in rats [88] and decrease the odds of heavy drinking by 45% in a randomized clinical trial in humans [89]. Given that both addiction and schizophrenia GWASes have suggested an important role for *PDE4B* in disease risk, PDE4B inhibitors may also benefit schizophrenia patients. A Phase I study in 15 schizophrenia patients found that roflumilast, an inhibitor of all four *PDE4* phosphodiesterases, significantly improved verbal memory, but not working memory [90].

We prioritized *VRK2*, which encodes vaccinia-related kinase 2 (Fig. 3F). While the role of *VRK2* in schizophrenia remains unclear, it is expressed in microglial cells and a mechanism involving synaptic elimination by microglial cells has been proposed [91, 92]. Like *PDE4B*, the same addiction GWAS [46] also found an association near *VRK2*. The addiction and schizophrenia signals colocalize (H_4_ = 92%), suggesting a shared causal variant. Therefore, modulating VRK2 activity might result in clinical benefit for people with SUD and/or schizophrenia. VRK2 is a member of the highly-druggable serine/threonine kinases group of enzymes [58] and has been co-crystallised with a small molecule ligand [93]. *VRK2* modulation could be tested in rodent addiction models and, if successful, may warrant further testing in human clinical trials of SUD and SCZ patients.

Three other prioritized genes reside in loci shared with the addiction GWAS [46]: *DRD2*, *SLC39A8* (H_4_ = 100%), and *PLCL2* (H_4_ = 74%). Although our analyses did not find evidence that *SLC39A8* and *PLCL2* are easily druggable by small molecule drugs, knockdown or overexpression of these genes in rodent addiction models may nevertheless improve our understanding of the shared biology of addiction and schizophrenia.

### *GABBR2*

We prioritized *GABBR2*, which encodes the gamma-aminobutyric acid (GABA) type B receptor and is known to inhibit neuronal activity via downstream signaling cascades (Fig. 3D). A Phase II clinical trial is currently testing whether arbaclofen, a GABA_B_ receptor agonist, can rescue ASD symptoms [94]. Both post-mortem and in vivo studies identified reduced GABA levels in schizophrenia patients compared to controls, and impaired gamma band oscillations—which are linked with GABAergic signaling—are associated with schizophrenia [95–99]. If proven to be a successful therapy for ASD, arbaclofen may therefore represent an interesting drug repurposing candidate for schizophrenia, particularly for symptoms and socio-cognitive deficits that are shared between the two disorders [100, 101].

### *AKT3*

We prioritized *AKT3*, the member of the AKT serine/threonine-protein kinase gene family with the highest brain-specific expression. Capivasertib—an inhibitor of all three AKT kinases—was recently approved by the FDA to treat a subset of breast cancer patients [102]. However, AKT inhibition can lead to adverse psychiatric side effects [103] and *AKT3* knockout or knockdown resulted in cognitive deficits and reduced brain size in mice [104, 105]. Further studies are necessary to determine whether overall or isoform-specific [54] increases in AKT3 activity would benefit schizophrenia patients without increasing cancer risk.

### *SNCA*

We prioritized *SNCA*, which encodes α-synuclein (α-syn). α-syn aggregates are the pathological hallmark of Parkinson’s disease (PD) and antibodies targeting aggregated α-syn have been tested in two Phase II clinical trials for PD, although neither meet their primary endpoint [106, 107]. The schizophrenia association near *SNCA* colocalizes (H_4_ = 85%) with an association from a recent European-ancestry PD GWAS [47]. The schizophrenia risk allele was associated with increased PD risk, which is in turn linked to increased α-syn production [108]. As such, interventions that decrease α-syn production may benefit both PD and schizophrenia patients.

Besides *AKT3* and *SNCA*, there are 4 other genes that are targeted by approved or investigational drugs but have not been investigated in clinical trials of any psychiatric disorders. These include *CD47, KCNJ3, DPYD* and *ATP2A2*.

### Limitations

The PGC3 study prioritized 83 genes that were not prioritized in our study. The majority of these (49 genes) were prioritized via SMR. We did not include SMR because it only demonstrated a precision of 29% when predicting a “gold standard” dataset of causal and non-causal trait-gene pairs [28], consistent with recent models for systematic differences between variants highlighted by GWAS and expression studies [109]. The precision of SMR-nominated genes that failed to meet our gene prioritization criteria is therefore likely to be lower than 29%. Only one additional gene (*CLCN3*) had SMR evidence and the top PoPS value in its locus. The PGC3 study prioritized an additional 26 genes where the entire credible set resided within the gene body, but did not have the top PoPS value. These included 6 non-coding genes: *AC068490.2, CTD-2008L17.2, LINC00320, LINC01088, RP11-399D6.2*, and *RP11-507B12.2*. The original PoPS study estimated that nearest genes without PoPS evidence had a < 27% probability of being a “probable causal gene” [28]. In the PoPS study, selecting the top-ranked gene in a locus led to a higher precision than including all genes above a specific threshold [28]. While we nominated only one gene per locus, some loci may contain multiple causal genes.

The original PGC3 study performed gene prioritization analyses in the “core dataset”. This excluded individuals of African (AFR) or Latin American (LAT) ancestry found in the “extended dataset”. To ensure consistency with the original PGC3 study, we also analyzed the core dataset. Furthermore, the AFR and LAT datasets only included GWAS summary statistics, not individual-level genotypes, preventing us from identifying well-matched LD reference panels—something particularly important for admixed populations [110]. Nevertheless, we stress the importance of expanding gene prioritization to include more ancestries to ensure that findings are generalizable to a broader range of people.

Finally, we assembled our list of prioritized schizophrenia genes using in silico methods. Future functional validation studies are critical for dissecting the biological processes underpinning schizophrenia risk and for selecting candidate targets for drug development or repurposing.

## Conclusion

We have curated a high-quality list of 101 genes that likely play a role in the development of schizophrenia. Developing or repurposing drugs that target these genes may lead to a new generation of schizophrenia therapies. Reassuringly, we prioritized several genes targeted by drugs that have been approved for schizophrenia (*DRD2*) or shown promising results in clinical trials (*e.g*., *GRIN2A*, *CACNA1C*, *CACN2B*). We prioritized genes that likely also play a role in SUD (*e.g*., *PDE4B*, *VRK2*). Drugs that modulate the activity of these genes could be tested in high-quality rodent models of addiction and, if shown to be safe and effective, should be considered for human clinical trials for SUD and/or schizophrenia. We also prioritized 7 genes that have not been targeted in clinical trials, but are predicted to be druggable via small molecule drugs. Additional efforts that improve our understanding of how these genes influence schizophrenia risk on a molecular level may stimulate the initiation of new drug programs. As new drug modalities continue to be invented and refined, more genes will become druggable. We hope that our list of prioritized genes will ultimately facilitate the development of new medicines for people living with schizophrenia.

## Supplementary information

Supplementary material

Supplementary Figure 1

Supplementary Tables

## Data Availability

ChEMBL Database: https://www.ebi.ac.uk/chembl/, HRC reference release 1.1: https://ega-archive.org/datasets/EGAD00001002729, Gencode release 44: https://www.gencodegenes.org/human/release_44.html, OpenTargets platform: https://platform-docs.opentargets.org/, The PGC3 GWAS core dataset is available through the PGC data access portal: https://pgc.unc.edu/for-researchers/data-access-committee/data-access-portal/, Summary statistics of the PGC3 GWAS are freely available for download: https://pgc.unc.edu/for-researchers/download-results/

## Code availability

Custom code used in the presented study is stored at https://github.com/kheilbron/cojo_pipe and https://github.com/kheilbron/brett, Additional software and code: COJO: https://yanglab.westlake.edu.cn/software/gcta/#COJO coloc: https://github.com/chr1swallace/coloc MAGMA: https://cncr.nl/research/magma/ PLINK 1.9: https://www.cog-genomics.org/plink/ PoPS: https://github.com/FinucaneLab/pops PsyOPS: https://github.com/Wainberg/PsyOPS
