# Supplementary material for: Identifying drug targets for schizophrenia through gene prioritization
Source: Transl Psychiatry. 2026 Feb 4;16:102. doi: 10.1038/s41398-026-03813-0 (PMC12923709; doi:10.1038/s41398-026-03813-0)
Supplement: Supplementary file 1 — Supplementary material [file 41398_2026_3813_MOESM1_ESM.docx]

## Comparison with previous schizophrenia gene prioritization efforts

In addition, we sought to compare our prioritized genes with those highlighted by a diverse array of recent literature. We assembled a set of studies that prioritized schizophrenia genes using gene co-expression[^1,2^](https://paperpile.com/c/0ePEvx/mzfue+l03bj), expression quantitative trait loci mapping or TWAS[^3–5^](https://paperpile.com/c/0ePEvx/70lAA+WDjBy+Z6kVL), epigenome-wide association study[^5^](https://paperpile.com/c/0ePEvx/Z6kVL), massively parallel reporter assays[^6^](https://paperpile.com/c/0ePEvx/FAT21), and 3D genome architecture analysis[^7^](https://paperpile.com/c/0ePEvx/hudUo). For Bhattacharya *et al*. 2023[^3^](https://paperpile.com/c/0ePEvx/70lAA), we took the union of all schizophrenia genes in Supplementary Data 11 and 13 with P_FDR_ < 0.05 and within-locus permutation *P*_ACAT_ < 0.05. For Borcuk *et al.* 2023[^1^](https://paperpile.com/c/0ePEvx/mzfue), we took all genes in Table S3. For Deans *et al*. 2025[^5^](https://paperpile.com/c/0ePEvx/Z6kVL), we took the union of genes in Tables 1A and 2B, representing schizophrenia gene sets 1 and 2. For McAfee *et al*. 2023[^6^](https://paperpile.com/c/0ePEvx/FAT21), we took all genes in Table S6. For Pergola *et al*. 2023[^2^](https://paperpile.com/c/0ePEvx/l03bj), we took all genes in Table 1. For Rajarajan *et al*. 2022[^7^](https://paperpile.com/c/0ePEvx/hudUo), we took all genes in Supplementary Table 10 and 18. To focus on finding causal genes in GWAS loci, we removed genes falling outside of PGC3 GWAS loci.

## Full list of the members of the Schizophrenia Working Group of the Psychiatric Genomics Consortium

Vassily Trubetskoy, Antonio F Pardiñas, Georgia Panagiotaropoulou, Swapnil Awasthi, Tim B Bigdeli, Charlotte A Dennison, Lynsey S Hall, Max Lam, Oleksandr Frei, Alexander L Richards, Jakob Grove, Zhiqiang Li, Mark Adams, Ingrid Agartz, Elizabeth G Atkinson, Esben Agerbo, Mariam Al Eissa, Margot Albus, Madeline Alexander, Behrooz Z Alizadeha, Köksal Alptekin, Thomas D Als, Farooq Amin, Volker Arolt, Manuel Arrojo, Lavinia Athanasiu, Maria Helena Azevedo, Silviu A Bacanu, Nicholas J Bass, Martin Begemann, Richard A Belliveau, Judit Bene, Beben Benyamin, Sarah E Bergen, Giuseppe Blasi, Julio Bobes, Stefano Bonassi, Alice Braun, Rodrigo Affonseca Bressan, Evelyn J Bromet, Richard Bruggeman, Peter F Buckley, Randy L Buckner, Jonas Bybjerg-Grauholm, Wiepke Cahn, Murray J Cairns, Monica E Calkins, Vaughan J Carr, David Castle, Stanley V Catts, Kimberley D Chambert, Raymond CK Chan, Boris Chaumette, Wei Cheng, Eric FC Cheung, Siow Ann Chong, David Cohen, Angèle Consoli, Quirino Cordeiro, Javier Costas, Charles Curtis, Michael Davidson, Kenneth L Davis, Lieuwe de Haan, Franziska Degenhardt, Lynn E DeLisi, Ditte Demontis, Faith Dickerson, Dimitris Dikeos, Timothy Dinan, Srdjan Djurovic, Jubao Duan, Giuseppe Ducci, Johan G Eriksson, Lourdes Fañanás, Stephen V Faraone, Alessia Fiorentino, Andreas Forstner, Josef Frank, Nelson B Freimer, Menachem Fromer, Alessandra Frustaci, Ary Gadelha, Giulio Genovese, Elliot S Gershon, Marianna Giannitelli, Ina Giegling, Paola Giusti-Rodríguez, Stephanie Godard, Jacqueline I Goldstein, Javier González Peñas, Ana González-Pinto, Srihari Gopal, Jacob Gratten, Michael F Green, Tiffany A Greenwood, Olivier Guillin, Sinan Gülöksüz, Raquel E Gur, Ruben C Gur, Blanca Gutiérrez, Eric Hahn, Hakon Hakonarson, Vahram Haroutunian, Annette M Hartmann, Carol Harvey, Caroline Hayward, Frans A Henskens, Stefan Herms, Per Hoffmann, Daniel P Howrigan, Masashi Ikeda, Conrad Iyegbe, Inge Joa, Antonio Julià, Anna K Kähler, Tony Kam-Thong, Yoichiro Kamatani, Sena Karachanak-Yankova, Oussama Kebir, Matthew C Keller, Brian J Kelly, Andrey Khrunin, Sung-Wan Kim, Janis Klovins, Nikolay Kondratiev, Bettina Konte, Julia Kraft, Michiaki Kubo, Vaidutis Kučinskas, Zita Ausrele Kučinskiene, Agung Kusumawardhani, Hana Kuzelova-Ptackova, Stefano Landi, Laura C Lazzeroni, Phil H Lee, Sophie E Legge, Douglas S Lehrer, Rebecca Lencer, Bernard Lerer, Miaoxin Li, Jeffrey Lieberman, Gregory A Light, Svetlana Limborska, Chih-Min Liu, Jouko Lönnqvist, Carmel M Loughland, Jan Lubinski, Jurjen J Luykx, Amy Lynham, Milan Macek Jr, Andrew Mackinnon, Patrik KE Magnusson, Brion S Maher, Wolfgang Maier, Dolores Malaspina, Jacques Mallet, Stephen R Marder, Sara Marsal, Alicia R Martin, Lourdes Martorell, Manuel Mattheisen, Robert W McCarley, Colm McDonald, John J McGrath, Helena Medeiros, Sandra Meier, Bela Melegh, Ingrid Melle, Raquelle I Mesholam-Gately, Andres Metspalu, Patricia T Michie, Lili Milani, Vihra Milanova, Marina Mitjans, Espen Molden, Esther Molina, María Dolores Molto, Valeria Mondelli, Carmen Moreno, Christopher P Morley, Gerard Muntané, Kieran C Murphy, Inez Myin-Germeys, Igor Nenadić, Gerald Nestadt, Liene Nikitina-Zake, Cristiano Noto, Keith H Nuechterlein, Niamh Louise O'Brien, F Anthony O'Neill, Sang-Yun Oh, Ann Olincy, Vanessa Kiyomi Ota, Christos Pantelis, George N Papadimitriou, Mara Parellada, Tiina Paunio, Renata Pellegrino, Sathish Periyasamy, Diana O Perkins, Bruno Pfuhlmann, Olli Pietiläinen, Jonathan Pimm, David Porteous, John Powell, Diego Quattrone, Digby Quested, Allen D Radant, Antonio Rampino, Mark H Rapaport, Anna Rautanen, Abraham Reichenberg, Cheryl Roe, Joshua L Roffman, Julian Roth, Matthias Rothermundt, Bart PF Rutten, Safaa Saker-Delye, Veikko Salomaa, Julio Sanjuan, Marcos Leite Santoro, Adam Savitz, Ulrich Schall, Rodney J Scott, Larry J Seidman, Sally Isabel Sharp, Jianxin Shi, Larry J Siever, Kang Sim, Nora Skarabis, Petr Slominsky, Hon-Cheong So, Janet L Sobell, Erik Söderman, Helen J Stain, Nils Eiel Steen, Agnes A. Steixner-Kumar, Elisabeth Stögmann, William S Stone, Richard E Straub, Fabian Streit, Eric Strengman, T Scott Stroup, Mythily Subramaniam, Catherine A Sugar, Jaana Suvisaari, Dragan M Svrakic, Neal R Swerdlow, Jin P Szatkiewicz, Thi Minh Tam Ta, Atsushi Takahashi, Chikashi Terao, Florence Thibaut, Draga Toncheva, Paul A Tooney, Silvia Torretta, Sarah Tosato, Gian Battista Tura, Bruce I Turetsky, Alp Üçok, Arne Vaaler, Therese van Amelsvoort, Ruud van Winkel, Juha Veijola, John Waddington, Henrik Walter, Anna Waterreus, Bradley T Webb, Mark Weiser, Nigel M Williams, Stephanie H Witt, Brandon K Wormley, Jing Qin Wu, Zhida Xu, Robert Yolken, Clement C Zai, Wei Zhou, Feng Zhu, Fritz Zimprich, Eşref Cem Atbaşoğlu, Muhammad Ayub, Alessandro Bertolino, Donald W Black, Nicholas J Bray, Gerome Breen, Nancy G Buccola, William F Byerley, Wei J Chen, C Robert Cloninger, Benedicto Crespo-Facorro, Gary Donohoe, Robert Freedman, Cherrie Galletly, Massimo Gennarelli, David M Hougaard, Hai-Gwo Hwu, Assen V Jablensky, Steven A McCarroll, Jennifer L Moran, Ole Mors, Preben B Mortensen, Bertram Müller-Myhsok, Amanda L Neil, Merete Nordentoft, Michele T Pato, Tracey L Petryshen, Ann E Pulver, Thomas G Schulze, Jeremy M Silverman, Jordan W Smoller, Eli A Stahl, Debby W Tsuang, Elisabet Vilella, Shi-Heng Wang, Shuhua Xu, Rolf Adolfsson, Celso Arango, Bernhard T Baune, Sintia Iole Belangero, Anders D Børglum, David Braff, Elvira Bramon, Joseph D Buxbaum, Dominique Campion, Jorge A Cervilla, Sven Cichon, David A Collier, Aiden Corvin, Marta Di Forti, Enrico Domenici, Hannelore Ehrenreich, Valentina Escott-Price, Tõnu Esko, Ayman H Fanous, Anna Gareeva, Micha Gawlik, Pablo V Gejman, Michael Gill, Stephen J Glatt, Vera Golimbet, Kyung Sue Hong, Christina M Hultman, Steven E Hyman, Nakao Iwata, Erik G Jönsson, René S Kahn, James L Kennedy, Elza Khusnutdinova, George Kirov, James A Knowles, Marie-Odile Krebs, Claudine Laurent-Levinson, Jimmy Lee, Todd Lencz, Douglas F Levinson, Qingqin S Li, Jianjun Liu, Anil K Malhotra, Dheeraj Malhotra, Andrew McIntosh, Andrew McQuillin, Paulo R Menezes, Vera A Morgan, Derek W Morris, Bryan J Mowry, Robin M Murray, Vishwajit Nimgaonkar, Markus M Nöthen, Roel A Ophoff, Sara A Paciga, Aarno Palotie, Carlos N Pato, Shengying Qin, Marcella Rietschel, Brien P Riley, Margarita Rivera, Dan Rujescu, Meram C Saka, Alan R Sanders, Sibylle G Schwab, Alessandro Serretti, Pak C Sham, Yongyong Shi, David St Clair, Ming T Tsuang, Jim van Os, Marquis P Vawter, Daniel R Weinberger, Thomas Werge, Dieter B Wildenauer, Xin Yu, Weihua Yue, Peter A Holmans, Panos Roussos, Evangelos Vassos, Danielle Posthuma, Ole A Andreassen, Kenneth S Kendler, Michael J Owen, Naomi R Wray, Mark J Daly, Hailiang Huang, Benjamin M Neale, Patrick F Sullivan, Stephan Ripke, James TR Walters, Michael C O'Donovan

## References

1 [Borcuk C, Parihar M, Sportelli L, Kleinman JE, Shin JH, Hyde TM *et al.* Network-wide risk convergence in gene co-expression identifies reproducible genetic hubs of schizophrenia risk. *Neuron* 2024; **112**: 3551–3566.e6.](http://paperpile.com/b/0ePEvx/mzfue)

2 [Pergola G, Parihar M, Sportelli L, Bharadwaj R, Borcuk C, Radulescu E *et al.* Consensus molecular environment of schizophrenia risk genes in coexpression networks shifting across age and brain regions. *Sci Adv* 2023; **9**: eade2812.](http://paperpile.com/b/0ePEvx/l03bj)

3 [Bhattacharya A, Vo DD, Jops C, Kim M, Wen C, Hervoso JL *et al.* Isoform-level transcriptome-wide association uncovers genetic risk mechanisms for neuropsychiatric disorders in the human brain. *Nat Genet* 2023; **55**: 2117–2128.](http://paperpile.com/b/0ePEvx/70lAA)

4 [Zeng B, Bendl J, Kosoy R, Fullard JF, Hoffman GE, Roussos P. Multi-ancestry eQTL meta-analysis of human brain identifies candidate causal variants for brain-related traits. *Nat Genet* 2022; **54**: 161–169.](http://paperpile.com/b/0ePEvx/WDjBy)

5 [Deans PJM, Townsley KG, Li A, Seah C, Johnson J, Gonzalez JG *et al.* Convergent and non-additive impact of schizophrenia risk genes in human neurons. bioRxiv. 2022; : 2022.03.29.486286.](http://paperpile.com/b/0ePEvx/Z6kVL)

6 [McAfee JC, Lee S, Lee J, Bell JL, Krupa O, Davis J *et al.* Systematic investigation of allelic regulatory activity of schizophrenia-associated common variants. *Cell Genom* 2023; **3**: 100404.](http://paperpile.com/b/0ePEvx/FAT21)

7 [Rajarajan P, Borrman T, Liao W, Schrode N, Flaherty E, Casiño C *et al.* Neuron-specific signatures in the chromosomal connectome associated with schizophrenia risk. *Science* 2018; **362**. doi:](http://paperpile.com/b/0ePEvx/hudUo)[10.1126/science.aat4311](http://dx.doi.org/10.1126/science.aat4311)[.](http://paperpile.com/b/0ePEvx/hudUo)
