# Supplementary material for: Identifying drug targets for schizophrenia through gene prioritization
Source: Transl Psychiatry. 2026 Feb 4;16:102. doi: 10.1038/s41398-026-03813-0 (PMC12923709; doi:10.1038/s41398-026-03813-0)
Supplement: Supplementary file 2 — Supplementary Figure 1 [file 41398_2026_3813_MOESM2_ESM.docx]

**
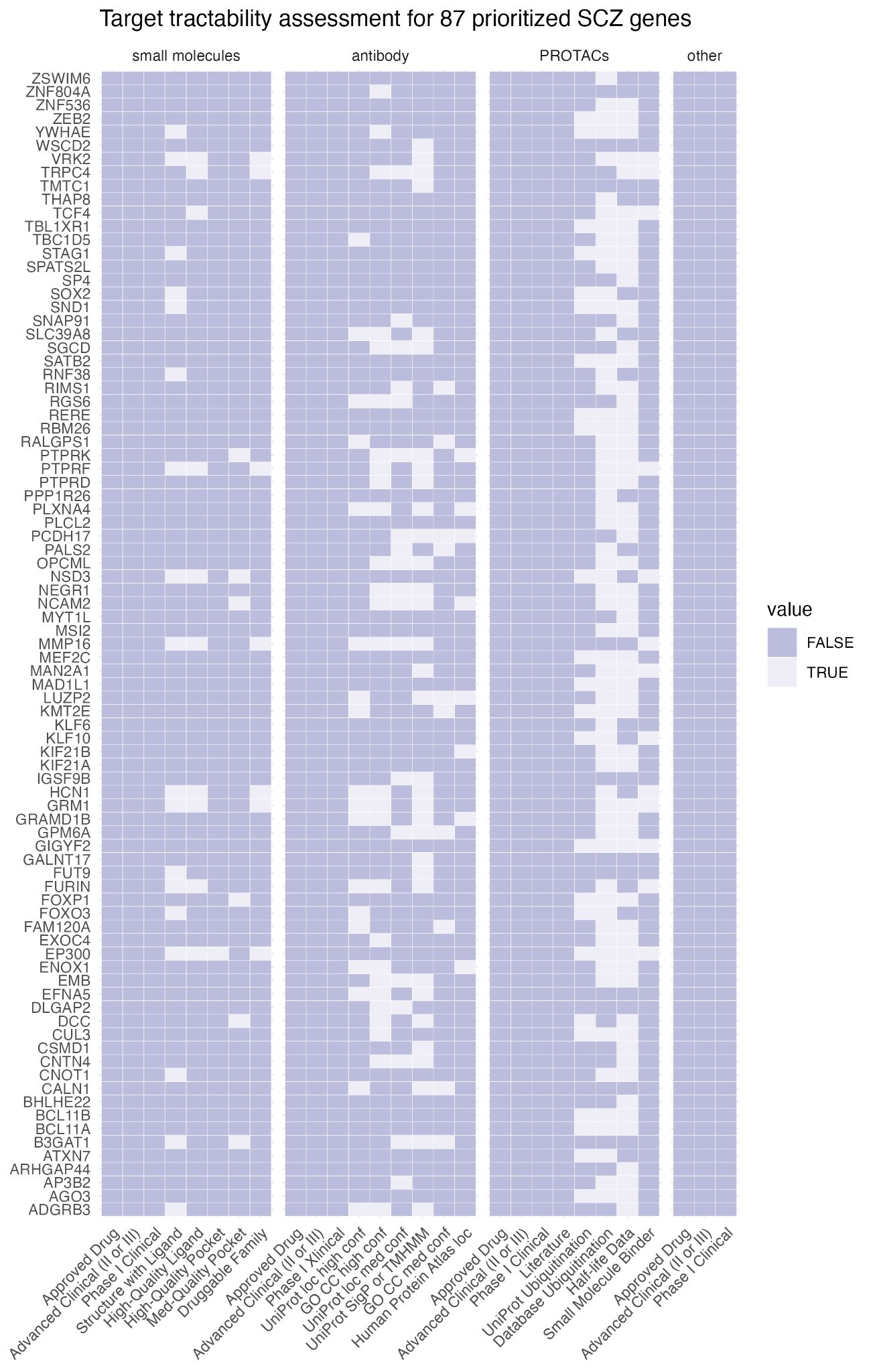
**

**Supplementary Figure 1**. Tractability of 87 prioritized schizophrenia (SCZ) genes (excluding 10 genes targeted by approved or investigational drugs) as novel drug targets by different modalities: small molecules, antibody, Proteolysis Targeting Chimeras (PROTACs) and other clinical modalities. Data was extracted from the Open Targets platform using GraphQL API queries (see Methods). Tractability buckets (x-axis) are presented in descending order of evidence quality within each modality.
